# Prospective evaluation of return to work, health-related quality of life and psychosocial distress after radical cystectomy: 1-year follow-up in 230 employed German bladder cancer patients

**DOI:** 10.1007/s00345-023-04570-1

**Published:** 2023-09-13

**Authors:** Guido Müller, Marius Cristian Butea-Bocu, Burkhard Beyer, Karl Heinrich Tully, Sebastian Berg, Florian Roghmann, Joachim Noldus, Henning Bahlburg

**Affiliations:** 1 Department of Urology, Marien Hospital Herne, Ruhr-University Bochum, Hölkeskampring 40, 44625 Herne, Germany; 2 Center for Urological Rehabilitation, Kliniken Hartenstein, Bad Wildungen, Germany

**Keywords:** Return to work, Quality of life, Psychosocial distress, Radical cystectomy, Bladder cancer

## Abstract

**Purpose:**

To evaluate return to work (RTW), health-related quality of life (HRQoL) and psychosocial distress (PD) after radical cystectomy (RC) and creation of an ileal conduit (IC) or an orthotopic ileal neobladder (NB) for bladder cancer.

**Methods:**

The study relied on prospectively collected data for 842 patients, who underwent 3 weeks of inpatient rehabilitation (IR) after surgery between April 2018 and December 2019. HRQoL (EORTC QLQ-C30) and PD (Questionnaire on Stress in Cancer Patients [QSC-R10]) were evaluated at the beginning (T1) and end (T2) of IR as well as both 6 (T3) and 12 months after surgery (T4). Regression analyses were performed to identify predictors of HRQoL and RTW, respectively.

**Results:**

Two hundred thirty patients (IC *n* = 51, NB *n* = 179) were employed before surgery (27.3%). HRQoL improved steadily, while high PD was present in 51.0% of patients at T4. RTW rate was 86.8 and 80.6% at T3 and T4, respectively. Linear regression analysis identified RTW as the only predictor for better HRQoL at T4 (OR [odds ratio] 12.823, 95% CI [confidence interval] 2.927–22.720, *p* = 0.012). Multivariate regression analysis identified age ≤ 59 years (OR 7.842; 95% CI 2.495–24.645; *p* < 0.001) as an independent positive predictor and lymph node metastasis (OR 0.220; 95% CI 0.054–0.893; *p* = 0.034) as an independent negative predictor of RTW at T4.

**Conclusion:**

Global HRQoL improved steadily during the follow-up and RTW rates are high. However, patients often reported high PD, reflecting a need for additional psychosocial support within aftercare.

## Introduction

For employed patients, return to work (RTW) after cancer treatment signals successful disease management and reintegration into the family and social environment [1]. The ileal conduit (IC) and orthotopic ileal neobladder (NB) are the most common types of urinary diversion after radical cystectomy (RC) for non-metastatic muscle-invasive bladder cancer [2–4]. Health-related quality of life (HRQoL) in patients after RC and urinary diversion is mostly reported in retrospective surveys from tertiary care centers, which is associated with certain limitations [5]. So that patients can reach the important goal of reintegration into normal life, German social laws entitle cancer patients to receive an average of three weeks of inpatient rehabilitation (IR). Especially for working patients, IR provides a comprehensive framework for a facilitated RTW. The guideline of the German Society of Urology recommends that all patients be offered several weeks of IR after RC for bladder cancer to minimize functional disorders, reduce psychosocial distress (PD) and improve HRQoL [6]. Porro et al. advocate that studies evaluating RTW in cancer survivors should focus on the first year after diagnosis [7]. This prospective, multi-institutional study allows us to report on HRQoL, PD and RTW in a large number of employed bladder cancer patients after RC and urinary diversion.

## Methods

### Study population

This prospective study is based on clinical data of patients with urothelial carcinoma of the bladder who underwent RC and IC or NB creation in various hospitals across Germany. All patients further underwent IR in a specialized center for urological rehabilitation (Kliniken Hartenstein, Bad Wildungen, Germany) between April 2018 and December 2019. The study protocol was approved by the appropriate ethics committee (research authorization number FF30/2017).

### Inpatient rehabilitation (IR)

During IR, patients were routinely seen twice a week by urological physicians and were treated daily by specialized nurses and physiotherapists regarding urinary continence and stoma care, respectively. Psychosocial interventions were performed by psychologists and social worker in addition to urologic counseling. The program includes information on bladder cancer and aftercare, individual, group and couple psychotherapy, relaxation training and psychoeducation.

### Variables, data collection and outcome measures

Baseline characteristics comprised patient age, Karnofsky performance status, body mass index (BMI), the existence of cardiovascular diseases and/or diabetes, tumor stage, method of surgery, utilization of neoadjuvant chemotherapy and socioeconomic status [8].

HRQoL and PD were evaluated at the beginning (T1) and end (T2) of IR as well as both 6 (T3) and 12 months (T4) after surgery. The use of individual psychotherapy during IR was recorded. HRQoL was assessed using the standardized European Organization for Research and Treatment of Cancer questionnaire (EORTC QLQ-C30) [9]. Normative data on the quality of life of the German general population were used for comparison [10]. The Questionnaire on Stress in Cancer Patients (QSC-R10) is a standardized and validated 10-item self-assessment instrument covering the main psychosocial aspects of cancer patients' daily life [11]. Response categories for each situation range from 0 (“not applicable”) to 5 (“it is a very serious problem”). The QSC-R10 total score is calculated by adding up the single items (≥ 15 demonstrates a high level of PD). Patients’ subjective positive prognosis regarding RTW was evaluated at T1 and T2. Data on work status were collected at T3 and T4.

### Statistical analyses

Descriptive analyses were preformed to examine sample characteristics and to describe RTW outcomes. Between-group comparisons were analyzed using the Mann–Whitney *U* test or Chi-square test (Pearson) as appropriate. The Wilcoxon test and the Chi-square test (McNemar) were used to assess the level of significance regarding changes during follow-up in quantitative variables and proportions. Regression analyses were performed to identify predictors for HRQoL and RTW. Significance was considered at *p* < 0.05. Analyses were performed with IBM SPSS version 27.

## Results

### Baseline characteristics and prospective follow-up

A total of 842 patients underwent RC in 135 different hospitals in Germany. Two hundred and thirty patients (IC *n* = 51, NB *n* = 179) were employed before surgery (27.3%, median age 58 years (IQR [interquartile range] 55–62)). The majority of patients were men (83.0%) with a NB rate of 85.9%, whereas women (17.0%) were more likely to receive an IC (61.5%). Low socioeconomic status was significantly more common in patients with IC (64.6%) than in patients with NB (40.0%). There were no group differences between the two types of urinary diversion with respect to Karnofsky performance status (median 80%), BMI (median 25 kg/m^2^) and the proportion of cardiovascular disease (42.2%), diabetes mellitus (6.5%), neoadjuvant chemotherapy (15.7%) or robotic surgery (11.7%). IC recipients had advanced tumor stage (≥ pT3: 43.1% vs. 22.9%) compared to NB patients. Lymph node metastases were found in 23.5% of IC patients and in 13.6% of patients with a NB—however, without a significance level (Table 1).

**Table 1:** Baseline characteristics of 230 employed patients after radical cystectomy

Variable	Total	Conduit	Neobladder	p*
Patients, *n* (%)	230 (100.0)	51 (22.2)	179 (77.8)	
Age (years), Median (IQR)	58 (55–62)	61 (57–62)	58 (55–61)	0.099
Gender, *n* (%)				
Male	191 (83.0)	27 (52.9)	164 (91.6)	**< 0.001**
Female	39 (17.0)	24 (47.1)	15 (8.4)	**< 0.001**
Karnofsky performance status (%), Median (IQR)	80 (70–80)	80 (70–80)	80 (70–80)	0.508
BMI (kg/m^2^), Median (IQR)	25 (23–28)	25 (21–29)	25 (23–27)	0.741
≥ 30, *n* (%)	26 (11.3)	9 (17.6)	17 (9.5)	0.105
Cardiovascular disease, *n* (%)	97 (42.2)	22 (43.1)	75 (41.9)	0.875
Diabetes, *n* (%)	15 (6.5)	1 (2.0)	14 (7.8)	0.135
Socioeconomic status, *n* (%)**				
Low	101 (45.3)	31 (64.6)	70 (40.0)	**0.002**
Middle	95 (42.6)	16 (33.3)	79 (45.1)	0.143
High	27 (12.1)	1 (2.1)	26 (14.9)	**0.016**
Neoadjuvant chemotherapy, *n* (%)	36 (15.7)	9 (17.6)	27 (15.1)	0.657
Method of surgery, *n* (%)				
Robot-assisted cystectomy	27 (11.7)	6 (11.8)	21 (11.7)	0.995
Open cystectomy	203 (88.3)	45 (88.2)	158 (88.3)	0.995
Tumor stage, *n* (%)				
≥ pT3a	63 (27.4)	22 (43.1)	41 (22.9)	**0.004**
Lymph node positive, *n* (%)***	35 (15.9)	12 (23.5)	23 (13.6)	0.090
No. of lymph nodes removed, Median (IQR)	17 (11–24)	16 (12–24)	18 (11–24)	0.762

Significant results (*p*<0.05) are shown in bold

*IQR* interquartile range, *BMI* body mass index

*Mann–Whitney *U* test or Chi-square test (Pearson) as appropriate

**Data available for 223 patients (conduit *n* = 48 and neobladder *n* = 175)

***Data available for 220 patients (conduit *n* = 51 and neobladder *n* = 169)

IR started at a median of 28 days (IQR 23–35) and ended with a median of 54 days (IQR 48–62) after surgery. Nobody died during IR. At T3, 14 patients (6.1%) had died, and in the period between T3 and T4, another 11 patients (4.8%) died. The response rates for the follow-up survey were 81.7% and 67.8% at T3 and T4, respectively. Fourteen and fifteen patients did not report their work at T3 and T4, respectively.

### Quality of life

At T1, HRQoL was significantly reduced in all functioning scales (Table 2). During IR, all scores of the different functioning scales significantly increased in IC and NB patients alike. Physical functioning scores were significantly higher in NB patients than in IC patients at the beginning of IR as well as at T3 and T4. After IR, there were further but less dynamic improvements in global HRQoL. Patients' emotional function temporarily decreased at T3. IC and NB patients did not differ in global HRQoL or role, emotional, cognitive and social functioning at T4. However, only the mean values of physical and cognitive functioning were comparable to the general German population at T4. Linear regression analysis identified RTW as the only predictor for better global HRQoL at T4 (OR [odds ratio] 12.823, 95% CI [confidence interval] 2.927–22.720, *p* = 0.012). Urinary diversion, age, sex, tumor stage or lymph node metastases were not predictors in this model.

**Table 2:** QLQ-C30 functional scales in patients who were employed prior to surgery

Variable	Total Mean (SD)	Conduit Mean (SD)	Neobladder Mean (SD)	p*
Global health status/quality of life				
T1	41.0 (20.1)	38.6 (17.3)	41.7 (20.8)	0.402
*p*^#^	** < 0.001**	** < 0.001**	** < 0.001**	
T2	55.0 (20.1)	57.8 (18.3)	54.3 (20.5)	0.313
*p*^#^	** < 0.001**	**0.224**	** < 0.001**	
T3	63.1 (21.1)	62.4 (21.4)	63.2 (21.0)	0.993
*p*^#^	**0.004**	0.779	** < 0.001**	
T4	65.5 (21.3)	62.0 (23.4)	66.5 (20.8)	0.381
Physical functioning				
T1	62.2 (20.8)	56.2 (16.3)	63.9 (21.6)	**0.017**
*p*^#^	** < 0.001**	**0.017**	**0.002**	
T2	67.0 (18.3)	62.7 (17.5)	68.2 (18.4)	0.075
*p*^#^	** < 0.001**	0.083	** < 0.001**	
T3	76.9 (19.7)	69.5 (20.6)	78.9 (19.0)	**0.004**
*p*^#^	**0.010**	0.784	**0.003**	
T4	80.5 (19.6)	73.3 (19.5)	81.9 (19.3)	**0.013**
Role functioning				
T1	32.3 (33.8)	39.4 (30.5)	33.1 (34.7)	0.716
*p*^#^	** < 0.001**	**0.005**	** < 0.001**	
T2	45.6 (32.0)	47.8 (28.2)	45.1 (33.0)	0.612
*p*^#^	** < 0.001**	0.637	** < 0.001**	
T3	56.7 (30.0)	52.2 (33.4)	57.9 (29.1)	0.369
*p*^#^	**0.028**	0.832	**0.009**	
T4	61.5 (30.8)	54.7 (32.3)	63.3 (30.3)	0.175
Emotional functioning				
T1	54.3 (27.6)	47.8 (26.0)	56.2 (27.9)	**0.043**
*p*^#^	** < 0.001**	** < 0.001**	** < 0.001**	
T2	70.2 (25.9)	66.1 (23.7)	71.3 (26.4)	0.103
*p*^#^	** < 0.001**	**0.030**	** < 0.001**	
T3	61.7 (27.2)	58.8 (30.6)	62.4 (26.3)	0.619
*p*^#^	**0.005**	0.562	** < 0.001**	
T4	65.0 (28.2)	58.4 (32.8)	66.7 (26.7)	0.206
Cognitive functioning				
T1	74.7 (23.9)	74.5 (23.4)	74.8 (24.1)	0.898
*p*^#^	** < 0.001**	** < 0.001**	** < 0.001**	
T2	83.0 (21.0)	87.2 (15.6)	81.9 (221)	0.280
*p*^#^	**0.001**	**0.008**	**0.024**	
T3	78.9 (22.4)	77.9 (22.6)	79.1 (22.5)	0.732
*p*^#^	0.094	0.321	0.154	
T4	81.2 (22.1)	80.2 (22.2)	81.5 (22.2)	0.734
Social functioning				
T1	45.6 (29.2)	49.3 (29.8)	44.6 (29.0)	0.260
*p*^#^	** < 0.001**	**0.001**	** < 0.001**	
T2	58.9 (28.7)	66.7 (26.2)	56.8 (29.1)	**0.031**
*p*^#^	0.329	**0.043**	0.905	
T3	58.4 (30.9)	58.5 (32.9)	58.4 (30.5)	0.999
*p*^#^	**0.025**	0.154	0.064	
T4	61.4 (29.5)	63.0 (29.6)	61.0 (29.6)	0.648

Significant results (*p*<0.05) are shown in bold

*T1* beginning of inpatient rehabilitation (28 days (IQR 23–35) after surgery), *T2* end of inpatient rehabilitation (54 days (IQR 48–62) after surgery), *T3* 6 months after surgery, *T4* 12 months after surgery, *SD* standard deviation

*Mann–Whitney *U* test

^#^Wilcoxon test

### Psychosocial distress

At T1, the study cohort reported high PD with a median score of 16 (IQR 9.5–23). PD decreased significantly during IR to a median score of 11 (IQR 4–18), but increased significantly at both T3 and T4 to a median score of 14.5 (IQR 6–26) and 15 (IQR 7–22), respectively (Table 3). The portion of patients suffering from high PD decreased significantly from 55.3% at T1 to 38.5% at T2 (*p* < 0.001). Unfortunately, the portion of patients reporting high PD increased to 50.0% at T3 and remained elevated at 51.0% at T4. No significant differences were detected when comparing patients with IC or NB in terms of PD. Nonetheless, individual psychotherapy during IR was more frequently used by IC patients (IC 58.8% vs. NB 41.3%; *p* = 0.027). Regression analyses failed to identify predictors of high PD after IR.

**Table 3:** Psychosocial distress (QSC-R10) in patients who were employed prior to surgery

Variable	Total	Conduit	Neobladder	p*
Total score				
T1, Median (IQR)	16 (9.5–23)	15 (11–25)	16 (9.5–23)	0.515
*p*^#^	** < 0.001**	**0.002**	** < 0.001**	
T2, Median (IQR)	11 (4–18)	12 (6–18)	10 (4–18.5)	0.222
*p*^#^	** < 0.001**	**0.037**	** < 0.001**	
T3, Median (IQR)	14.5 (8–26)	14 (9–25)	15 (8–26)	0.577
*p*^#^	**0.004**	0.210	**0.008**	
T4, Median (IQR)	15 (7–22)	17 (6.5–24)	13.5 (7–22)	0.348
Cutoff ≥ 15				
T1, *n* (%)	126 (55.3)	28 (57.1)	98 (54.7)	0.765
*p*^#^	** < 0.001**	0.065	** < 0.001**	
T2, *n* (%)	87 (38.5)	21 (42.0)	66 (37.5)	0.564
*p*^#^	**0.005**	0.508	**0.009**	
T3, *n* (%)	85 (50.0)	16 (48.5)	69 (50.4)	0.846
*p*^#^	0.523	1.000	0.359	
T4, *n* (%)	75 (51.0)	19 (61.3)	56 (48.3)	0.198

Significant results (*p*<0.05) are shown in bold

*QSC-R10* questionnaire on stress in cancer patients (10 items), *T1* beginning of inpatient rehabilitation (data available for 228 patients), *T2* end of inpatient rehabilitation (data available for 226 patients), *T3* 6 months after surgery (data available for 170 patients), *T4* 12 months after surgery (data available for 147 patients), *IQR* interquartile range

*Mann–Whitney *U* test or Chi-square test (Pearson) as appropriate

^#^Wilcoxon test or Chi-square test (McNemar) as appropriate

### Return to work

Patients’ subjective positive prognosis regarding RTW was 85.7% and 77.4% at T3 and T4, respectively. At both time points, NB patients were significantly more optimistic regarding RTW compared to IC patients (89.4% vs. 72.5%; *p* < 0.001 and 82.1% vs. 60.8%; *p* < 0.001). At T3, patients with a subjectively positive prognosis regarding RTW reported better global HRQoL and lower PD than patients with a subjectively negative prognosis regarding RTW (global HRQoL: mean 57.3 (SD 18.8) vs. 46.4 (SD 22.5); *p* = 0.005; QSC-R10: median 9 (IQR 4–17) vs. 17 (IQR 9.5–24); *p* < 0.001).

RTW rate was 86.8% (*n* = 151) and 80.6% (*n* = 116) at T3 and T4, respectively. At T4, 9.0% of patients (*n* = 13) retired, 12 patients (8.3%) were unemployed, and 2.1% (*n* = 3) received a disability pension. Of those employed at T4, 84.5% (*n* = 98) worked full-time. At T4, patients with RTW had better global HRQoL than patients without RTW (mean 69.8 (SD 18.9) vs. 55.1 (SD 25.4); *p* = 0.004).

Univariate logistic regression analysis identified age ≤ 59 years (OR 6.967; 95% CI 2.613–18.572; *p* < 0.001), NB (OR 2.579; 95% CI 1.008–6.595; *p* = 0.048), tumor stage ≤ pT2 (OR 3.235; 95% CI 1.278–8.189; *p* = 0.013) and lymph node metastasis (OR 0.242; 95% CI 0.080–0.729; *p* = 0.012) as predictors for RTW at T4. Age ≤ 59 years (OR 7.842; 95% CI 2.495–24.645; *p* < 0.001) was identified as an independent positive predictor, while lymph node metastases (OR 0.220; 95% CI 0.054–0.893; *p* = 0.034) were identified as an independent negative predictor in multivariate regression analysis for RTW at T4, and male sex trended toward significance (*p* = 0.051). Surgical technique and high socioeconomic status did not influence RTW (Table 4).

**Table 4:** Logistic regression analysis to identify predictors for return to work at 12 months after radical cystectomy

	Univariate		Multivariate	
	OR (95% CI)	*p*	OR (95% CI)	*p*
Age ≤ 59 years (yes vs. no)	6.967 (2.613–18.572)	** < 0.001**	7.842 (2.495–24.645)	** < 0.001**
Male vs. female	2.641 (0.941–7.411)	0.065	4.834 (0.992–23.567)	0.051
Neobladder vs. Conduit	2.579 (1.008–6.595)	**0.048**	0.813 (0.205–3.221)	0.768
Robotic vs. open cystectomy	2.080 (0.450–9.625)	0.349	1.224 (0.217–6.901)	0.819
Tumor stage ≤ pT2 (yes vs. no)	3.235 (1.278–8.189)	**0.013**	2.570 (0.707–9.340)	0.152
Positive nodal stage (yes vs. no)	0.242 (0.080–0.729)	**0.012**	0.220 (0.054–0.893)	**0.034**
High socioeconomic status (yes vs. no)	2.553 (0.558–11.687)	0.227	2.123 (0.399–11.293)	0.377

Significant results (*p*<0.05) are shown in bold

*OR* odds ratio, *CI* confidence interval

## Discussion

Employment is associated with a higher HRQoL in cancer patients. Self-assessed work ability is an important factor in the process of cancer patients RTW [1]. The study of Andreu et al. showed that employment had the greatest positive association with HRQoL. Younger patients and singles were identified as high-risk groups for an impaired HRQoL in cancer survivors [12]

In the current study, global HRQoL improved steadily during follow-up. However, compared with the population-based sample, only the mean values of physical and cognitive functioning were equal one year after surgery. In this cohort, surgical approach (open vs robot-assisted RC) did not influence RTW. Two recently published studies have compared HRQoL in patients undergoing robot-assisted RC and open RC. Venkatramani et al. could not show a difference in the recovery of activities of daily life 1 year after RC. The randomized controlled trial of Mastroianni et al. reported a significant impairment of role functioning, symptom scales and bowel symptoms in patients receiving open RC, while patients after robot-assisted RC reported significant impairment of urinary symptoms and problems 1 year after surgery [13, 14]. In addition to impaired HRQoL, 51.0% of patients in our study presented high PD at T4. Linear regression analysis identified RTW as the only predictor for improved global HRQoL at T4. RTW rate was 80.6% at T4. Multivariate regression analysis identified age ≤ 59 years and absence of lymph node metastases as predictors of RTW at T4.

In Germany, the current RTW rate of oncological patients is 61–64% [15, 16]. The incidence of cancer in Germany has increased significantly over the last 30 years. Among current 55-year-olds, 47.3% of men and 38.8% of women are expected to develop cancer within the next 10 years. However, the 5-year relative survival rate across all tumor entities is increasing and was 65% for women and 59% for men in 2016 [17]. If this trend continues, measures to reintegrate tumor patients into working life will become increasingly important in the context of the already evident shortage of skilled workers [18]. Occupational reintegration is influenced by the type of tumor disease, oncological therapy, physical and psychological limitations, comorbidity, patient's motivation, as well as sociodemographic and work-related factors [19, 20]. After RC with creation of an urinary diversion, there is an increased risk of limitation of the ability to work, due to therapy-related changes in physical functioning. Long-term cancer survivors have an unemployment rate of 34% (compared to 15% in the healthy comparison group) [21]. The likelihood of RTW is higher if there is a good social network at the workplace and if the employer both creates favorable working conditions and offers assistance in RTW [22]. Possible long-term adverse effects of (neo)adjuvant chemotherapy include peripheral neuropathic paresthesia and motor impairment, chronic myelosuppression with rapid fatigability and cognitive impairment. However, cognitive deficits are also commonly observed in cancer patients and affect attention, memory (especially short-term memory), verbal memory (word-finding problems) and the ability to solve complex tasks [23, 24]. On the other hand, cognitive stimulation and physical activity improve cognitive impairment, memory and attention [25]. Porro et al. described the multifactorial process of RTW and a theory-based clinical framework for interventions to promote RTW of cancer survivors. According to individual situations, support should be sufficiently flexible and provide a range of simple tools [26]. For the social system, the successful reintegration of patients into working life represents an essential dimension of the success of rehabilitation measures. A calculation based on the data of rehabilitants from 2010 shows that the costs of a rehabilitation measure amortize by 4 months of longer participation in working life due to saved disability pensions and collected contributions [18]

The current study reported continued improvement in HRQoL and a high rate of RTW for bladder cancer patients after RC and creation of a urinary diversion. However, patients often showed increasing PD after discharge from IR. In addition to urological cancer aftercare, patients should be referred to outpatient psycho-oncological care services and to self-help groups.

Despite its strengths, our study has several limitations. We did not collect data on HRQoL and PD before surgery. No distinction was made between different types of hospitals and there was no control group outside the rehabilitation program. As inpatient rehabilitation is specific to the German healthcare system, results may not be generalizable to other healthcare systems.

## Conclusion

After RC, employed bladder cancer patients often reported significantly reduced HRQoL and high PD. During IR, all scores of the different HRQoL functioning scales improved and PD decreased significantly. After discharge from IR, patients showed further improvement in HRQoL and high rates of RTW. Nonetheless, PD was elevated in about 50% of patients. To stabilize patients in the long term, it seems to be important to inform patients about psychosocial support within aftercare.

## Data Availability

Not applicable.

## Abbreviations

BMI: Body mass index
CI: Confidence interval
EORTC: European Organization for Research and Treatment of Cancer
IC: Ileal conduit
IR: Inpatient rehabilitation
IQR: Interquartile range
HRQoL: Health-related quality of life
NB: Neobladder
OR: Odds ratio
PD: Psychosocial distress
QLQ-C30: Quality of Life Questionnaire (30 items)
QSC-R10: Questionnaire on Stress in Cancer Patients (10 items)
RC: Radical cystectomy
RTW: Return to work
T1: Beginning of inpatient rehabilitation
T2: End of inpatient rehabilitation
T3: 6 Months after radical cystectomy
T4: 12 Months after radical cystectomy
